# Methanogenesis coupled hydrocarbon biodegradation enhanced by ferric and sulphate ions

**DOI:** 10.1007/s00253-024-13278-0

**Published:** 2024-08-29

**Authors:** Krisztián Laczi, Attila Bodor, Tamás Kovács, Balázs Magyar, Katalin Perei, Gábor Rákhely

**Affiliations:** 1 Department of Biotechnology, University of Szeged, Szeged, Hungary; 2 Biological Research Centre, Institute of Plant Biology, Hungarian Research Network, Szeged, Hungary; 3 Biological Research Centre, Institute of Biophysics, Hungarian Research Network, Szeged, Hungary; 4 Department of Biotechnology, Nanophage Therapy Center, Enviroinvest Corporation, Pécs, Hungary; 5 Biocentrum Ltd, Gyöngyösoroszi, Heves Hungary

**Keywords:** Bioremediation, Oil, Methane, Aromatics, Aliphatics, Electron acceptors

## Abstract

**Abstract:**

Bioremediation provides an environmentally sound solution for hydrocarbon removal. Although bioremediation under anoxic conditions is slow, it can be coupled with methanogenesis and is suitable for energy recovery. By altering conditions and supplementing alternative terminal electron acceptors to the system to induce syntrophic partners of the methanogens, this process can be enhanced. In this study, we investigated a hydrocarbon-degrading microbial community derived from chronically contaminated soil. Various hydrocarbon mixtures were used during our experiments in the presence of different electron acceptors. In addition, we performed whole metagenome sequencing to identify the main actors of hydrocarbon biodegradation in the samples. Our results showed that the addition of ferric ions or sulphate increased the methane yield. Furthermore, the addition of CO_2_, ferric ion or sulphate enhanced the biodegradation of alkanes. A significant increase in biodegradation was observed in the presence of ferric ions or sulphate in the case of all aromatic components, while naphthalene and phenanthrene degradation was also enhanced by CO_2_. Metagenome analysis revealed that *Cellulomonas* sp. is the most abundant in the presence of alkanes, while *Ruminococcus* and *Faecalibacterium* spp. are prevalent in aromatics-supplemented samples. From the recovery of 25 genomes, it was concluded that the main pathway of hydrocarbon activation was fumarate addition in both *Cellulomonas*, *Ruminococcus* and *Faecalibacterium*. Chloroflexota bacteria can utilise the central metabolites of aromatics biodegradation via ATP-independent benzoyl-CoA reduction.

**Key points:**

• *Methanogenesis and hydrocarbon biodegradation were enhanced by Fe*^*3+*^* or SO4*^*2−*^

• *Cellulomonas, Ruminococcus and Faecalibacterium can be candidates for the main hydrocarbon degraders*

• *Chloroflexota bacteria can utilise the central metabolites of aromatics degradation*

**Graphical Abstract:**

**Figure:**
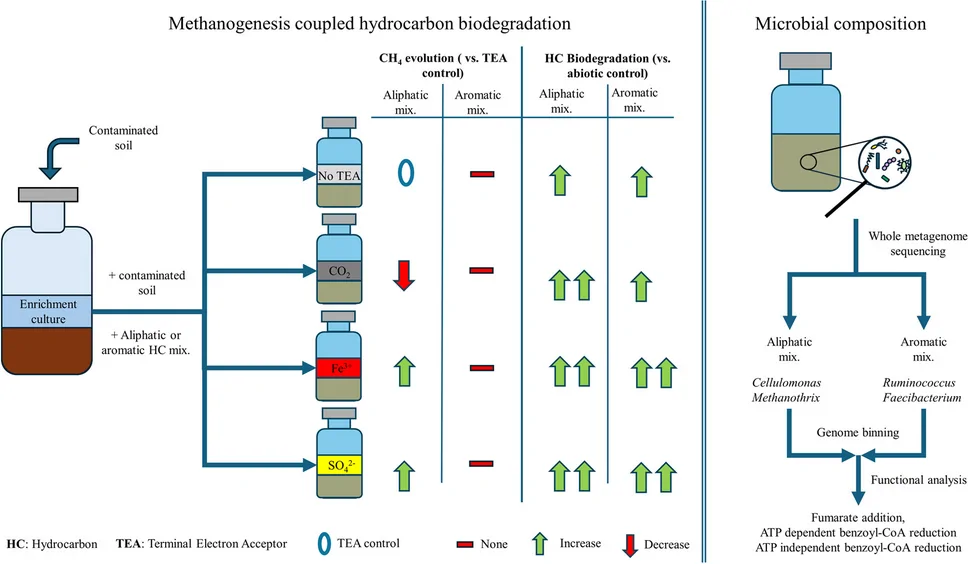

**Supplementary Information:**

The online version contains supplementary material available at 10.1007/s00253-024-13278-0.

## Introduction

Crude oil and its derivatives still supply a significant part of the energy worldwide. The extensive usage leads to oil spills, which contaminate the environment. Certain hydrocarbon compounds such as PAHs, BTEX and volatile alkanes imply a serious threat to human and animal health as well (Davidson et al. 2021; Sun et al. 2021; Halios et al. 2022). Larger spills such as the consequence of the BP Deepwater Horizon catastrophe in 2010 can also inflict significant damage on the economy of the affected area (Buckingham-Howes et al. 2019; Keating et al. 2020). The negative impact of hydrocarbons on the environment, health and economy is indisputable, and rapid intervention is essential.

Bioremediation can be an environmentally safe and cost-effective solution for the removal of hydrocarbons (da Silva et al. 2020). Many bacterial groups are capable of hydrocarbon biodegradation either under aerobic or anaerobic conditions (Abbasian et al. 2016; Laczi et al. 2020). The aerobic pathways are faster and more effective than their anaerobic counterparts, and they are preferred during bioremediation. However, hydrocarbons can seep into deeper soil layers or sediments where the environment is anoxic and hinders biodegradation. Bacteria developed enzymes including succinate synthases to activate hydrocarbons (Fuchs et al. 2011; Boll et al. 2014). Such enzymes, e.g. alkyl/benzyl-succinate synthases, occur in *Smithella* sp., *Thauera aromatica* and *Azoarcus* sp. (Leuthner and Heider 2000; Achong et al. 2001; Tan et al. 2014). Similar enzymes in the Asgard group of archaea were recently found, indicating their ability to utilise hydrocarbons (Zhang et al. 2021). The anaerobic mineralisation of aromatic compounds consists of a peripheral and a central pathway (Fuchs et al. 2011). Alternative activation pathways of the aromatic compounds, such as carboxylation and hydroxylation, are also known (Boll et al. 2014). The central pathway utilises benzoyl-CoA as the central metabolite of aromatics biodegradation. It can occur in an ATP-dependent and ATP-independent manner catalysed by Class I and Class II benzoyl-CoA reductases (Rabus et al. 2016; Tiedt et al. 2018; Anselmann et al. 2019).

Alternative terminal electron acceptors (TEA) including nitrate, sulphate and ferric ions sustain anaerobic respiration. Still, their presence can be scarce in deep soil or sediments as their concentration decreases with depth (Li et al. 2009). Anaerobic hydrocarbon biodegradation can also couple with methanogenesis. The methane produced from hydrocarbon biodegradation can be utilised as an energy carrier, partially recovering the energy of the oil spill (Parkes 1999; Xia et al. 2016; Suda et al. 2021). Microbially enhanced energy recovery can be used to decrease the costs of remediation.

In this study, we performed laboratory-scale batch fermentation experiments with hydrocarbon-contaminated soil samples derived from the B4 sampling site of the NATO base in Taszár, Hungary, from a depth of 7.5 m described earlier (Molnár et al. 2020). We examined the effect of sulphate and ferric ions on hydrocarbon biodegradation and biomethane production. We also determined the microbial community composition and recovered metagenome-assembled genomes (MAGs) from sequencing data.

## Materials and methods

### Sampling

The sample for the enrichment culture was taken from the B4 sampling site of the military base of Taszár from 7.5 to 8.0 m (Molnár et al. 2020). The hydrocarbon-contaminated soil was collected into 500-mL borosilicate bottles (Teqler, Wecker, Luxembourg) and sealed with a silicone septum. The bottle was flushed with nitrogen (purity: 5.0, Messer Hungarogas Ltd. Budapest, Hungary). Soil (30 g) was kept anaerobically and was immediately frozen at − 80 °C for later metagenomic analysis as the samples arrived at the lab.

### Starter enrichment culture

Enrichment cultures were prepared under anaerobic conditions in a Bactron IV™ Anaerobic Chamber (Sheldon Manufacturing Inc., Cornelius, OR, USA). The chamber’s atmosphere was set with a 5% hydrogen in 95% nitrogen gas mixture (Linde Gas Hungary Co CLtd., Budapest, Hungary). Fresh soils (30 g) were weighed into 160-mL serum bottles (Sigma-Aldrich, St. Louis, MO, USA) and supplemented with 30-mL heat-sterilised distilled water. The vials were sealed with butyl-PTFE septa (Sigma-Aldrich). The vials were flushed with nitrogen (purity: 5.0) and then incubated at 30 °C in an incubator without shaking. Head-space gas composition was measured weekly. Starter cultures were utilised after 6 weeks, where methane levels were stabilised in the head space.

### Preparation of the samples

**Fig. 1:**
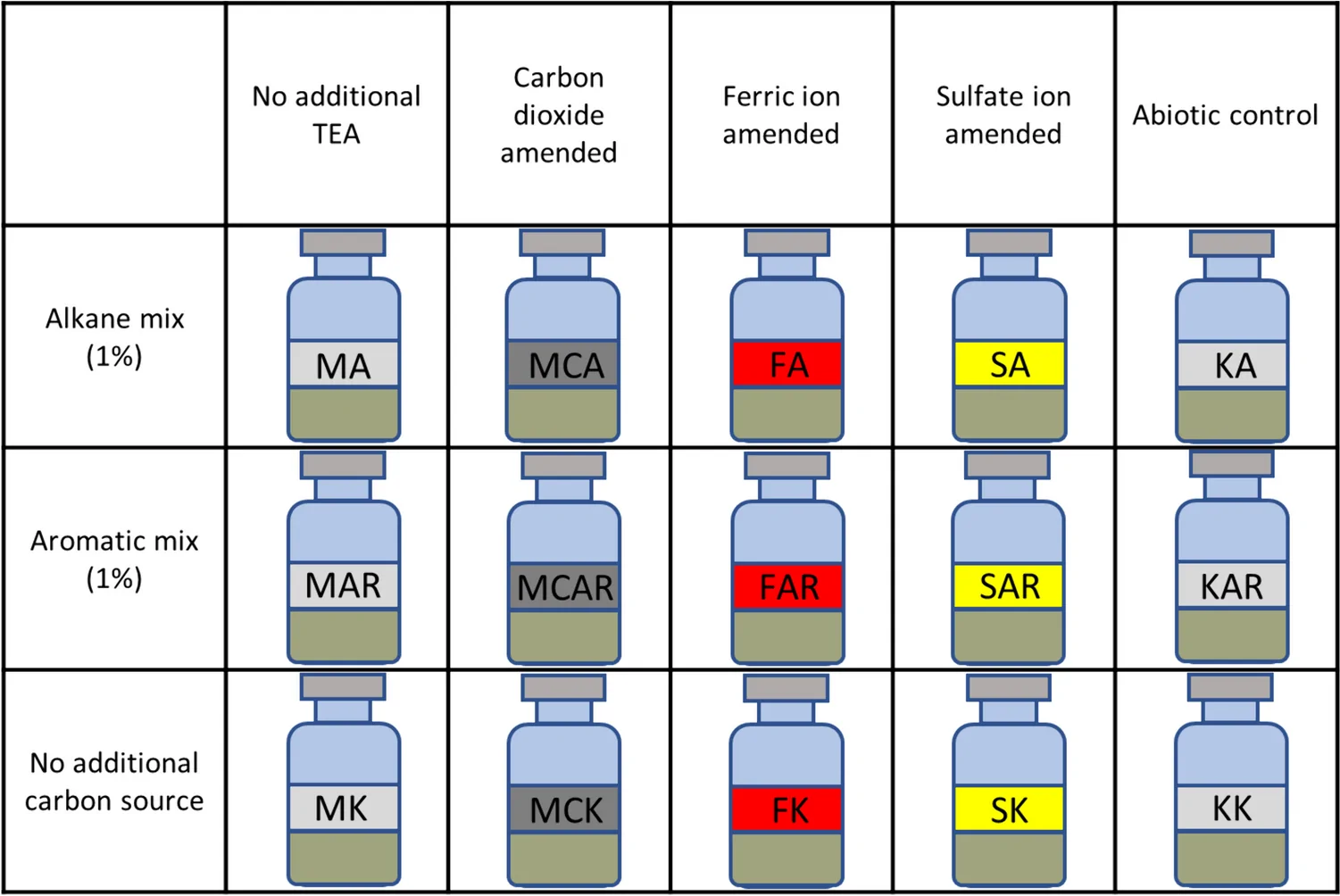
Composition of samples. The colours of the labels indicate the applied electron acceptor ferric ion (red), sulphate (yellow), carbon dioxide amended (dark grey) and no additional electron acceptor (light grey). The abbreviations of the labels are used across the article to indicate the samples

Samples were prepared in a Bactron IV™ Anaerobic Chamber. The three starter cultures were mixed, and 0.5-mL suspensions were aliquoted into serum bottles (full volume: 38 mL headspace: 30 mL), and then 5 g of the original soil was added to the samples. All samples were supplemented with a 4.5-mL minimal salt medium containing various electron acceptors (Fig. 1) to facilitate hydrocarbon biodegradation. Table 1 summarises the compositions of media used during the experiments. All samples were sealed with butyl-PTFE septa and flushed with nitrogen (purity: 5.0) for 5 min. After flushing, 6-mL nitrogen was removed from the CO_2_-amended samples with a syringe and an equal volume of food industrial CO_2_ (Gourmet C, Messer Hungarogas Ltd.) was added. This resulted in a concentration of 20% v/v in the headspace. Three types of carbon sources were added to each electron acceptor containing samples using a Hamilton glass syringe. An aliphatic mixture (61.6-mg n-hexadecane, 3.9-mg pristane, 3.9-mg cyclohexane, 3.9-mg methyl-cyclohexane and 3.6-mg n-decane) and an aromatic mixture (90-mg methyl-naphthalene, 4.3-mg toluene, 4.3-mg ethyl-benzene, 2.7-mg naphthalene and 2.5-mg phenanthrene). A sample group without an additional carbon source was prepared as a biotic control for each electron acceptor. Abiotic controls for GC/MS measurements were prepared as follows. A total of 5 g of original soils were heat sterilised with the Tyndall method with three sterilisation cycles. The heat-sterilised soils were mixed with a minimal salt medium lacking externally added electron acceptors. The samples were incubated at 30 °C in a stationary incubator. All samples were prepared in sextuplicates except for the biotic and abiotic control samples, which were only triplicated.

**Table 1:** Composition of the utilised media

Component	Sulphate amended	Iron amended	No additional TEA
K2HPO4 (mg/L), K2HPO4 (mg/L)	870	870	870
KH2PO4 (mg/L), KH2PO4 (mg/L)	680	680	680
NaCl (mg/L)	585	585	585
MgSO4 × 7H2O (mg/L), MgSO4 × 7H2O (mg/L)	125		
MgCl2 × 6H2O (mg/L), MgCl2 × 6H2O (mg/L)		102.5	102.5
CaCl2 × 2H2O (mg/L), CaCl2 × 2H2O (mg/L)	44	44	44
ZnSO4 × 7H2O (mg/L), ZnSO4 × 7H2O (mg/L)	0.2		
ZnCl2 (mg/L), ZnCl2 (mg/L)		0.094	0.094
MnCl2 (mg/L), MnCl2 (mg/L)	0.06	0.06	0.06
H3BO3 (mg/L), H3BO3 (mg/L)	0.6	0.6	0.6
CoCl2 × 6H2O (mg/L), CoCl2 × 6H2O (mg/L)	0.4	0.4	0.4
CuCl2 × 2H2O (mg/L), CuCl2 × 2H2O (mg/L)	0.02	0.02	0.02
NiCl2 × 6H2O (mg/L), NiCl2 × 6H2O (mg/L)	0.04	0.04	0.04
NaMoO4 × 4H2O (mg/L), NaMoO4 × 4H2O (mg/L)	0.046	0.046	0.046
FeSO4 (mg/L), FeSO4 (mg/L)	14		
FeCl3 × 6H2O (mg/L)		24.86	
FeCl2 × 4H2O (mg/L)			18.32
Na2EDTA × 2H2O (mg/L)	11.85	11.85	11.85
NH4Cl (mg/L)	802	802	802

### Analytical methods

#### Determination of methane concentration in the headspace

Methane concentration was measured with an Agilent 6890 gas chromatograph (Agilent Technologies Inc., Santa Clara, CA, USA) equipped with a split/splitless (S/SL) injector and a thermal conductivity detector (TCD). The analytical column was an HP-Molsieve (30 m × 0.53 mm × 25 μm) with a 5-Å pore size. The carrier gas was argon (purity 5.0, Messer Hungarogas Ltd.). Column flow was 1.1 mL/min, and the oven temperature was set to 47 °C. The injector was operated in split mode with a split ratio of 9:1. Samples were injected manually with a gastight syringe (Hamilton 1725 RN, Hamilton Co., Reno, NV, USA). Methane calibration was done with a gas mixture containing 5% (v/v) methane, 5% (v/v) carbon dioxide and 5% (v/v) hydrogen (Linde Gas Hungary Co. Cltd.). Calibration samples were on atmospheric pressure. The molarity of CH_4_ was calculated based on the calibration curve with the ideal gas law using atmospheric pressure. The energy content of the methane was determined by first calculating the mass of the produced gas based on its molarity and then multiplying this mass by the calorific value of methane, which is 55.5 MJ/kg.

With this method, hydrogen and oxygen can also be measured from the sample headspace simultaneously with methane.

#### Determination of carbon dioxide concentration in sample headspace

Carbon dioxide was measured with a Shimadzu 2010 gas chromatograph equipped with an S/SL injector and a TCD. The analytical column was an HP-PLOTQ (30 m × 0.32 mm × 20 μm) capillary column. The carrier gas was nitrogen (purity 5.0, Messer Hungarogas Ltd.). The column flow was set to 1.25 mL/min, and the split ratio was 4:1. Samples were injected manually with a gastight syringe (1702 RN, Hamilton Co.) Calibration was performed with the same gas mixture as in the case of methane.

#### Determination of hydrocarbon concentration in the samples

Half of the samples (3–3 parallel from each set) and the abiotic controls were extracted with 5-mL diethyl-ether overnight at room temperature with shaking at 200 rpm on a Biosan OS-20-orbitary shaker (SIA Biosan, Riga, Latvia). The organic phase was sampled with syringes to avoid opening the vials. The hydrocarbon contents of the samples were measured with an Agilent 7890B gas chromatograph mounted with an Agilent 5975C VL-MSD mass spectrometer and an Agilent 7683B autosampler (Agilent Technologies Inc.). The gas chromatograph was also equipped with an S/SL injector. The analytical column was an HP-5 ms Ultra Inert (30 m × 0.25 mm × 0.25 μl) capillary column, and the carrier gas was helium (purity: 6.0, Messer Hungarogas Ltd.). From all samples, 1 μl was injected into the inlet. The energy of the electron beam was set to 70 eV in the electron impact chamber, and the mass spectrometer was run in single ion monitoring mode. The gas flow rate, the GC’s heating profile and the selected ions varied depending on the utilised carbon source as follows.

##### Aliphatic mixture

The gas flow in the column was set to 3.0 mL/min, and the split ratio was 29:1. The initial column temperature was 40 °C kept for 5 min. The heating ramp was set to 5 °C/min with a final temperature of 220 °C sustained for 3 min. The analysed ions were the following: m/z = 57, 83 and 84.

##### Aromatic mixture

The GC programme runs with a 1.2 mL/min column gas flow and a 19:1 split ratio. The initial oven temperature was 45 °C, held up for 4 min. The heating ramp was adjusted to 15 °C/min, and the final temperature was 230 °C kept for 5 min. The measured ions were m/z = 91, 128, 142 and 178.

### Preparation of DNA samples

DNA preparations were done from 5 mL of the cultures. In the case of the original soil sample, 2.5 g of soil was used. Samples were with the QiaGen PowerBead Tubes derived from the RNeasy PowerSoil Mini Kit (QIAGEN N.V. Venlo, the Netherlands). The kit’s protocol was followed until the first precipitation of nucleic acids. The precipitated nucleic acids were dissolved in 50-µL AccuGENE™ molecular biology water (Lonza, Basel, Switzerland) and purified by 1% agarose gel electrophoresis to eliminate contaminants entirely. Gel slices containing genomic DNA were cut with sterile blades and put into 0.5-mL PCR tubes. The bottom of the tubes was previously lined up with a thin layer of glass wool and heat sterilised. The gel slices were deep-freezed at − 80 °C for 2 h. Afterwards, the bottom of the tubes was perforated with a hot steel needle, and the liquid content of the gel slices was centrifuged (900 × g, 10 min) into a 1.5-mL microcentrifuge tube. The samples were treated with phenol:chloroform:isoamyl alcohol (25:24:1, pH = 6.6; Ambion, Austin, TX, USA), and then nucleic acids were precipitated with ice-cold isopropanol for 30 min at − 20 °C. After centrifugation (15,000 × g, 30 min), the pellets were washed with ice-cold 70% ethanol. DNA was dissolved in AccuGENE™ molecular biology water and sent for whole-metagenome sequencing.

### Whole metagenome sequencing

Sequencing was performed by Delta Bio 2000 Ltd. (Szeged, Hungary). Briefly, the DNA library was prepared with Nextera XT DNA Library Preparation Kit (Illumina Inc., San Diego, CA, USA) and sequenced on a NextSeq 550 (Illumina Inc.) with a NextSeq 500/550 Mid Output Kit v2.5 through 300 cycles as it was instructed by the manufacturer.

### Bioinformatical methods

The GNU parallel (Tange 2011) and homemade bash scripts were used to automate processes on multiple samples. Visualisation of the data was performed with Microsoft Excel and the seaborn (Waskom 2021), Pandas (McKinney 2010) and Matplotlib (Hunter 2007) modules of Python 3. For cluster map generation, the distance matrices were calculated with the Bray–Curtis method built in the seaborn module.

### Quality check and assessment of the reads

Read quality was summarised with FastQC (Andrews 2010), followed by MultiQC (Ewels et al. 2016) analysis. Quality trimming was performed with Trimmomatic (Bolger et al. 2014) with the following parameters: HEADING:2, TRAILING:3, SLIDINGWINDOW:8:20 and MINLEN:100. Only surviving paired reads were considered in the subsequent analysis.

### Read-based taxonomy

The taxonomic composition of the samples was determined with Kaiju (Menzel et al. 2016). Unassigned reads were omitted from further analysis, and taxons that did not reach 1% of the total read count in the particular sample were also not considered. Read abundance was normalised as a percentage of the assigned read number for the given sample.

### Whole metagenome assembly and MAG recovery

The quality trimmed samples were assembled into one large co-assembly with MEGAHIT (Li et al. 2015). The assembly statistics were generated by QUAST (Gurevich et al. 2013). MetaBat 2 (Kang et al. 2015), MaxBin 2 (Wu et al. 2016) and Concoct (Alneberg et al. 2014) were used for binning of individual MAGs. Bin refinement of the MAGs was carried out with MetaWrap (Uritskiy et al. 2018). The Quant_bins module of MetaWrap was used to estimate the abundance of the individual MAGs through the samples. The MAG abundance results were expressed in genome copies per million reads (GCPM). Log_2_ GCPM values were visualised with the cluster map module of seaborn. The reassembled bins were identified with GTDB-tk (Chaumeil et al. 2020), and their functional analysis was performed with Prokka (Seemann 2014) and eggnog-mapper (Huerta-Cepas et al. 2017). Individual genes were identified with blastx and blastp algorithms using the MAGs and their deduced proteins against known proteins as a query.

### Statistical analysis

Statistical analysis was performed in SigmaPlot using one-way analysis of variance (ANOVA; *p* ≤ 0.05) followed by Duncan’s multiple range test.

## Results

### Methane and carbon dioxide evolution of the cultures

**Fig. 2:**
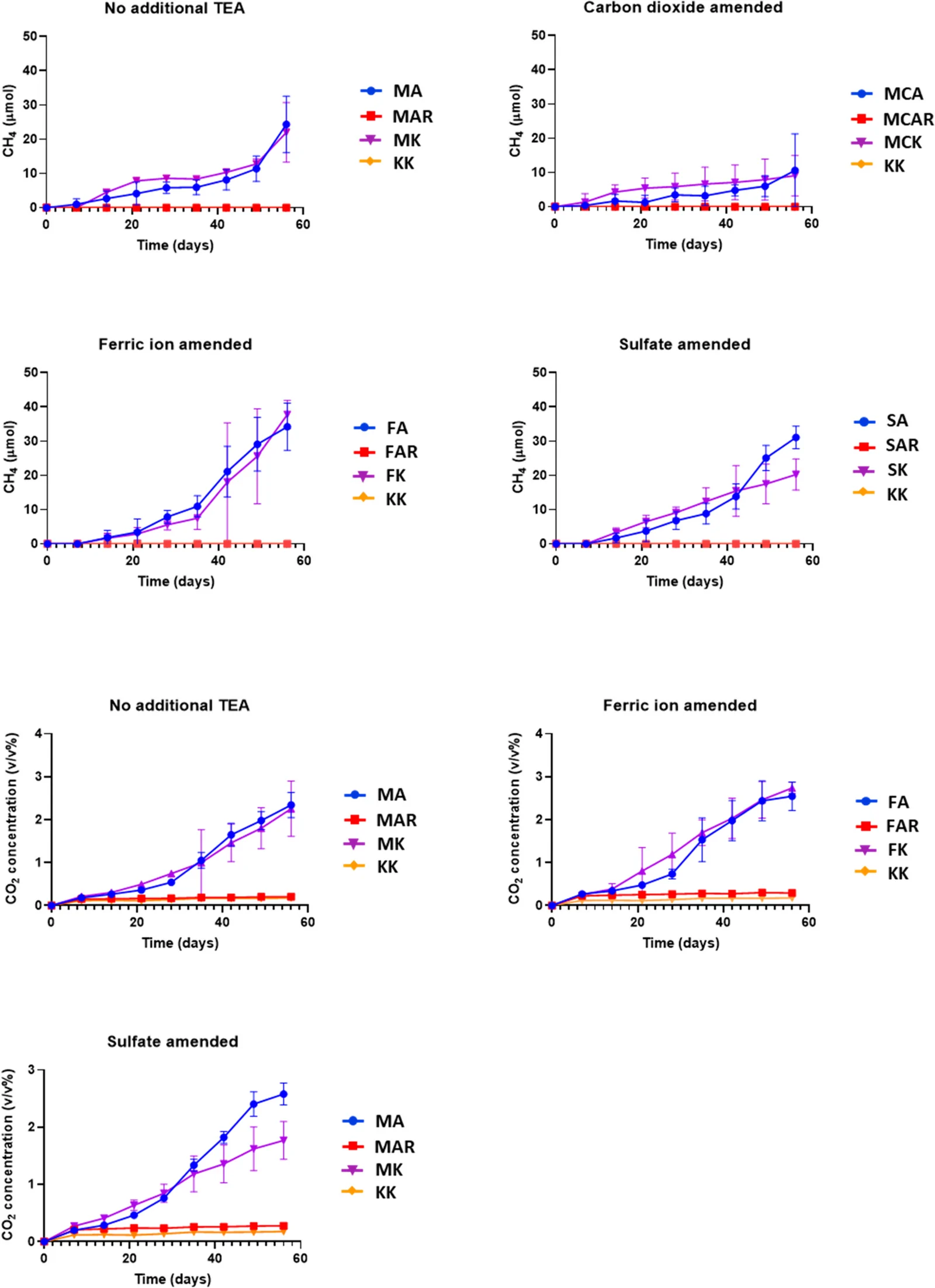
Methane and carbon dioxide evolution in the samples during the incubation period

**Fig. 3:**
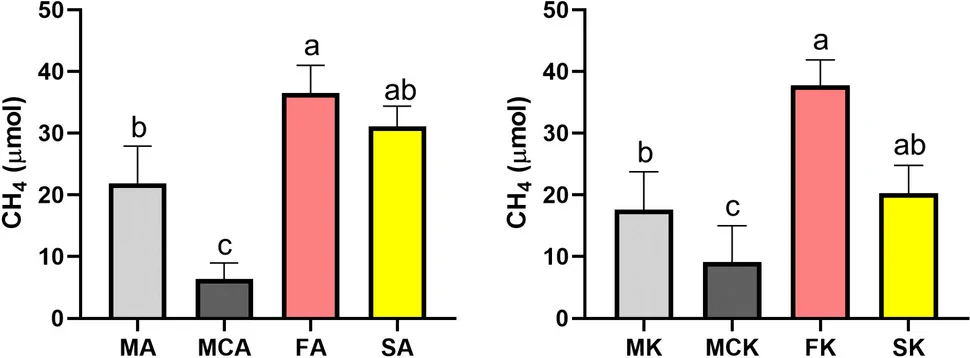
Methane yield of the alkane-amended samples (**A**) and their corresponding controls (**B**) at the end of the incubation period. Different letters indicate statistical differences among treatments according to Duncan’s test (*n* = 6, *p* ≤ 0.05)

We have followed methane evolution during the entire length of the incubation period. The measurements were performed weekly. All samples produced methane during the 8 weeks except for the samples amended with the aromatics mixture (FAR, MAR, MCAR, SAR) and the abiotic controls (KA, KAR, KK) (Fig. 2). In samples, where the methanogenesis was not inhibited, the methane concentration increased gradually as time passed. The fastest methane production was reached in the FK and FA samples. These samples produced 37.7 ± 4.1 µmol and 34.2 ± 6.9 µmol methane, respectively, until the end of the incubation period. FK and FA produced significantly more methane than their counterparts without additional electron acceptors MA and MK, which yielded only 24.4 ± 8.3 and 22.0 ± 8.7 µmol methane, respectively (Fig. 3). SA samples also produced high amounts of methane (31.1 ± 3.3 µmol). Although statistical significance was not found between SA and MA, the SA samples show tendentially higher methane yield (Fig. 3). The addition of alkanes showed only a slight increase in methane yields in the presence of sulphate compared to SK in the last 2 weeks. Carbon dioxide-containing samples (MCK and MCA) yielded less methane than their carbon dioxide-lacking counterparts (MK and MA). We also calculated the energy content of the produced methane. It was the highest in the FK samples 33.5 ± 3.7 J in total or 7.5 ± 0.8 J relative to 1 g of the original soil sample and the lowest in MCA 5.7 ± 2.3 J in total or 1.3 ± 0.5 J relative to the original soil (Table S1).

Hydrogen and oxygen concentrations were also followed but remained under the detection limit during the incubation period.

Carbon dioxide evolution in the samples was followed to determine the metabolic activity of the microbial community in the non-CO_2_-amended samples. CO_2_-amended samples contained an initial concentration of 20% (v/v) CO_2_, which was far over the linear range of our TCD detector and made it impossible to measure any increase or decrease of the CO_2_ concentration in the headspace. In KA, KAR and KK, an increase in carbon dioxide concentration was not detectable except for the first week when there was an initial increase from 0 to 0.1% (v/v). A slightly higher initial increase was observed in the aromatics mixture-amended samples (MAR, FAR, SAR), but the final concentration did not exceed 0.3% (v/v). Statistical analysis showed a significant difference between MAR, FAR, SAR and their corresponding abiotic control KAR on the last day of incubation. The other samples had a gradual increase in carbon dioxide, and the final concentration reached an average of 2.5% (v/v) except for the SK where a slight decrease was observable compared to the other samples with an average of 1.7% (v/v). This was in concordance with the methane concentrations of SK samples, where we observed a significant drop compared to that of SA.

### Bioconversion rate of hydrocarbons under different conditions

**Fig. 4:**
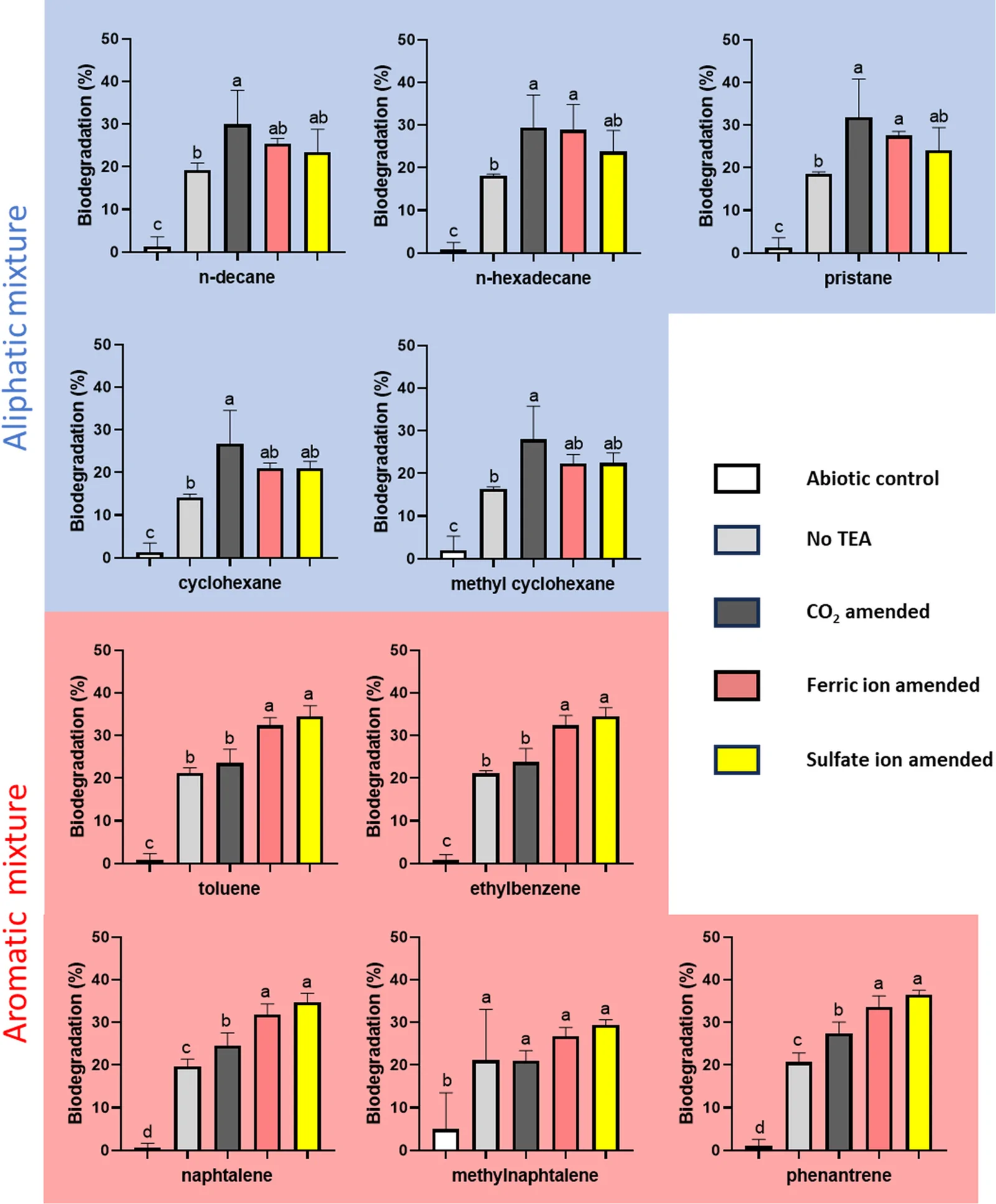
The biodegradation rate of individual hydrocarbon components at the end of the incubation period. Different letters indicate statistical differences among treatments according to Duncan’s test (*n* = 3, *p* ≤ 0.05)

The original soil contained weathered diesel contamination, which had been there for two decades. The concentration of the original contamination was 4500 mg/kg soil. We supplemented this indigenous carbon source with an aliphatic mixture in the case of MA, FA, SA and their abiotic control KA or aromatics mixture in the case of MAR, FAR, SAR and their abiotic control KAR. We measured the biodegradation rate on the last day of incubation for every component of the exogenous carbon source compared to their corresponding abiotic control (Fig. 4). There was a significant elevation of the biodegradation rate in all aliphatics amended samples compared to KA. Generally, the biodegradation of alkanes was the slowest in the MA samples (14.2–19.2% was degraded after 8 weeks) and the fastest in MCA samples (26.7–31.8%). Carbon dioxide addition significantly increased the biodegradation rate of alkanes and cycloalkanes compared to MA. We also observed an elevated biodegradation rate in the presence of ferric ions or sulphate. However, n-hexadecane biodegradation showed a significant elevation in the FA samples compared to MA.

The biodegradation of aromatics showed a different picture. Although neither carbon dioxide nor methane was generated in the aromatics mixture-amended sample, a substantial elevation in biodegradation of aromatics was observed in the MAR, MCAR, FAR and SAR samples compared to the KAR samples. The highest rate was shown in the SAR samples (29.5–36.4%), while the lowest was in MAR (19.7–21.1%). The biodegradation rate in FAR was only slightly lower than SAR, and both were significantly higher for all the components but methylnaphthalene compared to MAR and MCAR. The MAR and MCAR samples acted similarly on all components except for naphthalene and phenanthrene, which were degraded at a significantly higher rate in the presence of carbon dioxide.

### Shotgun metagenome sequencing

DNA was extracted from the samples, and whole metagenome sequencing was performed in triplicates. In total, 38,909,341 paired-end reads were generated with an average length of 135 bases. After quality trimming, 84.5% of the reads were preserved with an average length of 129 bases.

### Microbial composition of the enrichment cultures

**Fig. 5:**
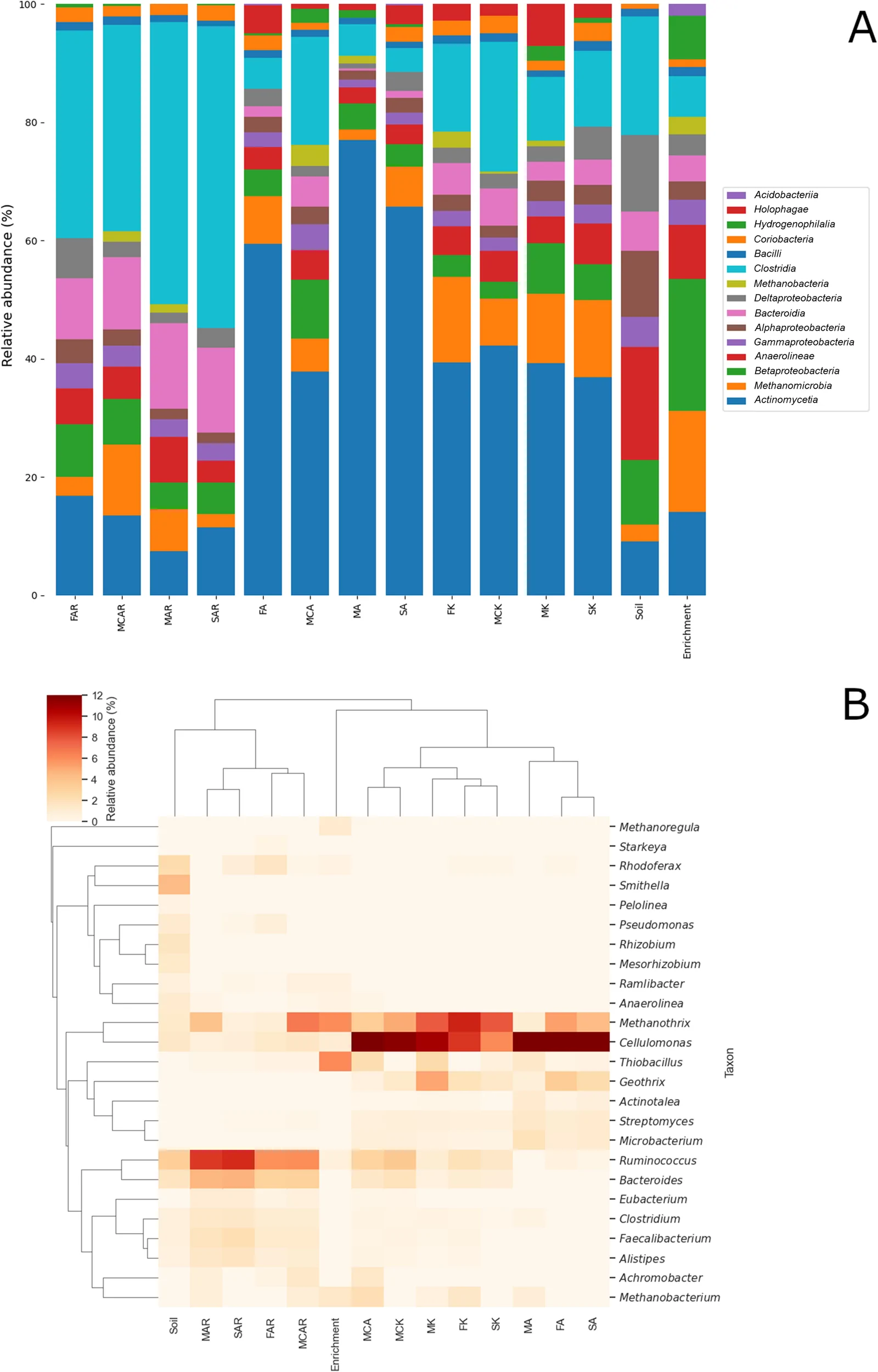
The abundance of bacterial and archaeal classes (**A**) Bray–Curtis clustermap of abundant microbial genera (**B**) calculated from sequencing reads with Kaiju

The microbial composition of every sample was determined by assigning the reads to taxons with Kaiju. A bar chart shows the abundances of various prokaryotic classes (Fig. 5a), and a cluster map was generated from the data on genus level (Fig. 5b). The major classes of the original soil appeared in all the samples, but their ratios changed based on the treatment groups. In the original soil, multiple classes are dominant: Clostridia (14.79%), *Anaerolineae* (13.95%), Deltaproteobacteria (9.41%), Alphaproteobacteria (8.20%), Betaproteobacteria (8.01%) and Actinomycetia (6.69%). In the enrichment culture, Betaproteobacteria (13.71%) and Methanomicrobia (10.48%) became the most dominant, followed by Actinomycetia (8.62%). In contrast, Actinomycetes were predominant in the alkane-amended samples (27.94–64.75%) and in the biotic controls (23.67–30.75%). The abundance of *Clostridia* (3.03–13.52%, 8.17–16.05%, respectively) and Methanomicrobia (1.45–5.94%, 5.77–10.20%, respectively) is also remarkable in these samples. In the aromatics-amended samples, Clostridia (24.04–37.20%) became the most dominant class, and Bacteroidia (7.07–10.54%) also emerged. Actinomycetia (5.39–11.72%) and Methanomicrobia (1.64–8.45%) remained also dominant, but their relative abundances were much lower compared to the alkane-amended samples, and the biotic controls stayed in the range of the original soil and the enrichment culture.

The samples are divided into two major clusters based on their microbial composition (Fig. 5B). The first cluster contains the aromatics-amended samples and the original soil. However, the original soil showed similarities in the microbial composition with the aromatics-amended samples. Its predominant genus, *Smithella* (formerly belonging to Deltaproteobacteria, reassigned to Desulfobacterota (Waite et al. 2020)), which might be the main hydrocarbon degrader in the contaminated soil, was completely missing from the other samples. Some other genera, like *Anaerolinea*, *Rhodoferax*, *Sphingopyxis*, *Rhizobium* and *Mesorhizobium*, were missing or only sporadically present across the samples. The main cause of this radical change in the microbial composition of the samples was the temperature difference between the deep soil and the laboratory experiment. This is supported by the substantial changes in the community structure of the enrichment culture compared to the original soil. The enrichment culture had two dominant genera, *Methanothrix* (6.02%) and *Thiobacillus* (6.09%). *Methanomicrobium* (1.53%), *Methanoregula* (1.25%) and *Cellulomonas* (1.00%) were also present.

*Ruminococcus* (5.89–9.16%) was predominant in the aromatics-amended cluster regardless of the type of TEA present (Fig. 5B). *Bacteroides* (3.03–4.78%) along with *Faecalibacterium* (1.31–2.23%) were also dominant genera in the aromatics-amended samples. *Alistipes* (0.91–1.78%), *Clostridium* (1.06–1.58%) and *Cellulomonas* (0.72–1.65%) are prevalent in the aromatics-amended samples. Even though active methanogenesis was not detected in the aromatics-amended samples, the genus *Methanothrix* (0.66–6.77%) was present in this cluster.

The second cluster contained the alkane-amended samples and the biotic controls. The enrichment culture belonged to this cluster. The genus *Cellulomonas* was predominant among the alkane-amended samples (16.36–32.45%) and in the majority of the biotic controls (6.07–11.63%), although it had lower relative abundance in the latter samples. *Streptomyces* and *Microbacterium* had a higher abundance in MA and SA samples compared to the corresponding biotic controls (MK, SK). In FA and MCA samples, there was no significant difference in the abundance of the two genera compared to the corresponding controls (FK, MCK). In addition, another member of the Cellulomonadaceae, *Actinotalea*, was only present in MA, FA and SA samples. *Geothrix* (0.62–5.15%) was present among the alkane-amended samples and their corresponding controls with the highest abundance in MK and the lowest in MCA.

*Methanothrix* was ubiquitous in this cluster of samples, but its abundance decreased in the presence of alkanes. Carbon dioxide also had a negative effect on the prevalence of the genus *Methanothrix*. Furthermore, in MA and MCA, as well as in MK and FK samples, *Methanobacterium* also appeared. This genus along with *Methanoregula* and *Methanothrix* composed the bulk of the methanogenic archaea in the enrichment culture.

### Genomes recovered from the microbial communities

We have co-assembled the reads of the alkane- and the aromatics-amended samples. The assembly resulted in 155,159 contigs with a minimum length of 200 bp. The N50 value was 1755 with the largest contig of 869,339 bp. A total of 90% of the reads could be mapped back to the assembly. Twenty-five metagenome-assembled genomes (MAGs) were recovered; out of them, 18 were high quality, and 7 were medium quality (Table 2) according to the MIMAG protocol (Bowers et al. 2017). Despite the high completeness and low contamination of the MAGs, only four of them could be identified on the species level and six others on the genus level. The other MAGs were identified on the family level or above. We have found 3 Euryarchaeota MAGs (bin 4, bin 11 and bin 18). All the 3 MAGs were methanogenic archaea, namely *Methanothrix* (bin 04), *Methanobacterium* (bin 11) and an unidentified genus (UBA288) of the family Methanosphaerulaceae (bin 18). This family was recently proposed, and *Methanosphaerula* was moved to it as a type genus from Methanoregulaceae (Rinke et al. 2021). The uncultivated Bacteria and Archaea (UBA) genera and other taxonomic groups were identified in a large genome recovery project (Parks et al. 2017). The *Methanoregula* sp. found in the read-based taxonomy belonged to this MAG since we used an older database with Kaiju created prior to the publication of the Rinke article.

**Table 2:** The characteristics and identity of the recovered MAGs

Bin name	Completeness	Contamination	GC	Lineage	N50	Size	Identification based on GTDB
bin.01	100	3.189	0.635	Betaproteobacteria	33,707	3,235,476	Bacteria, Proteobacteria, Gammaproteobacteria, Burkholderiales, Thiobacillaceae, *Thiobacillus*
bin.02	84.97	2.361	0.676	Actinobacteria	12,455	1,671,344	Bacteria, Actinobacteriota, Coriobacteriia, OPB41, PALSA-660, PALSA-660
bin.03	95.9	4.545	0.486	Bacteria	36,113	3,411,527	Bacteria, Chloroflexota, *Anaerolineae*, Anaerolineales, Anaerolineaceae
bin.04	97.38	2.124	0.518	Euryarchaeota	19,992	2,646,289	Archaea, Halobacteriota, *Methanosarcina*, Methanotrichales, *Methanotrichaceae*, *Methanothrix*
bin.05	98.65	11.32	0.734	Actinomycetales	47,151	3,194,694	Bacteria, Actinobacteriota, Actinomycetia, Actinomycetales, Cellulomonadaceae, *Cellulomonas*
bin.06	93.08	1.666	0.667	Actinobacteria	13,739	1,867,183	Bacteria, Actinobacteriota, Coriobacteriia, OPB41, UBA2279, UBA6100
bin.07	98.63	0	0.432	Clostridiales	103,835	2,637,760	Bacteria, *Firmicutes_A*, Clostridia, Oscillospirales, Ruminococcaceae, *Ruminococcus_D*, *Ruminococcus_D bicirculans*
bin.08	80.26	2.62	0.725	Actinomycetales	5623	2,921,308	Bacteria, Actinobacteriota, *Actinomycetia*, Actinomycetales, Cellulomonadaceae, *Cellulomonas*
bin.09	97.08	0.833	0.645	Actinobacteria	74,875	2,492,718	Bacteria, Actinobacteriota, Coriobacteriia, OPB41, UBA2279
bin.10	95.95	5.476	0.465	Bacteroidetes	6003	3,383,367	Bacteria, Bacteroidota, Bacteroidia, Bacteroidales, VadinHA17, LD21
bin.11	98.13	1.733	0.397	Euryarchaeota	11,311	2,429,629	Archaea, Methanobacteriota, Methanobacteria, Methanobacteriales, Methanobacteriaceae, Methanobacterium
bin.12	97.27	1.818	0.54	Bacteria	162,229	3,216,658	Bacteria, Chloroflexota, *Anaerolineae*, Anaerolineales, Anaerolineaceae, Bellilinea
bin.13	90.9	4.545	0.442	Bacteria	364,170	2,584,467	Bacteria, Chloroflexota, *Anaerolineae*, Anaerolineales, Anaerolineaceae, UBA4781
bin.14	76.41	0	0.33	Bacteria	16,235	797,038	Bacteria, Patescibacteria, Dojkabacteria, SC72, SC72
bin.15	95.3	1.677	0.413	Clostridiales	15,116	1,867,421	Bacteria, Firmicutes_A, Clostridia, Oscillospirales, Acutalibacteraceae, *Ruminococcus_E*, *Ruminococcus_E bromii*
bin.16	95.37	1.19	0.655	Bacteria	7506	2,606,247	*Bacteria, Desulfobacterota, MBNT15, MBNT15, MBNT15, CG2-30–66-27*
bin.17	89.79	1.845	0.447	Bacteria	5226	4,190,692	Bacteria, Bacteroidota; Bacteroidia, Bacteroidales, Prolixibacteraceae, UBA1413
bin.18	98.35	5.394	0.662	Euryarchaeota	23,248	2,978,862	Archaea, Halobacteriota, Methanomicrobia, Methanomicrobiales, Methanosphaerulaceae, UBA288, UBA288 sp11384u
bin.19	94.49	7.981	0.668	Bacteria	9506	3,813,631	Bacteria, Chloroflexota, *Anaerolineae*, UBA1429
bin.20	97.08	2.5	0.668	Actinobacteria	69,184	2,064,964	Bacteria, Actinobacteriota, Coriobacteriia, OPB41, UBA2279, UBA6100
bin.21	97.14	0	0.684	Bacteria	20,861	3,206,062	Bacteria, Acidobacteriota, Holophagae, Holophagales, Holophagaceae, *Geothrix*, *G. fermentans*
bin.22	94.03	1.834	0.627	Bacteria	43,256	2,644,479	Bacteria, Chloroflexota, Anaerolineae, UBA1429, UBA1429
bin.23	75.55	5.341	0.411	Clostridia	2522	2,994,698	Bacteria, Firmicutes_A, Clostridia, Lutisporales, Lutisporaceae, BRH-c25
bin.24	70.08	4.081	0.592	Clostridiales	2244	1,639,080	Bacteria, Firmicutes_A, Clostridia, Oscillospirales, Ruminococcaceae, *Faecalibacterium*,* F. prausnitzii*
bin.25	72.01	4.128	0.631	Bacteria	2079	2,306,881	Bacteria, Chloroflexota, Anaerolineae, UBA1429, UBA1429

The other 22 MAGs belonged to the Eubacteria kingdom. Two *Ruminococcus* species *Ruminococcus bromii* (bin 15) and *Ruminococcus bicirculans* (bin 7) along with *Faecalibacterium prausnitzii* (bin 24) and *Geothrix fermentans* (bin 21) were identified on the species level. On genus level *Thiobacillus* (bin 1), *Bellilinea* (bin 12), and two *Cellulomonas* (bins 5 and 8), MAGs were found. Many MAGs were assigned to UBA or other uncultivated genera or higher taxonomic levels.

The abundance of the MAGs in individual samples was calculated, and a cluster map was created from the data (Fig. S1). The clustering of the samples showed a similar pattern as in the case of the read-based abundance (Fig. 5B). Two major clusters were formed. One cluster contained the alkane-amended samples and the biotic controls, while the other cluster consisted of the aromatics-amended samples. MA was an outgroup in this case mainly due to the high abundance (average *GCPM* = 66.48) of bin 23. This was an unidentified genus BRH-c25 belonging to the family Lutisporaceae (Clostridia) and has an average nucleotide identity (ANI) of 80.08% to its closest relative BRH-c25 sp001515955. This microbe is not present in other samples of our experiments except for FK and SK but in a low average copy number of 3.55 and 4.55, respectively. The members of this genus have been found in rock porewater metagenome (PRJNA257561) and anaerobic digesters (PRJNA602310, PRJNA797469). The only publication so far proposed that a member of the genus BRH-c25 was capable of reductive alkane dehalogenation (Ning et al. 2022). Bin 05 (*Cellulomonas* sp.) has the highest abundance (2733.71 average GCPM) in the MA samples compared to other treatment groups (18.86–1224.38 average GCPM). Bin 5 had a high abundance among the alkane-amended samples and the biotic controls, while it decreased in the aromatic-amended samples. This was in concordance with the read-based results. The other *Cellulomonas* MAG (bin 08) had a low abundance across the samples. Therefore, bin 05 might be the main actor during alkane biodegradation. Our assumption was supported by the elevated abundance of bin 05 in the MA, FA and SA samples compared to their corresponding biotic controls MK, FK and SK. Another *Actinomycetota* MAG belonging to the UBA 6100 genus was prevalent in this sample cluster. The MAG shares 83.66% ANI with its closest relative UBA6100 sp002423585 which has been found in the metagenome of Syncrude tailings pond (Alberta, Canada), a heavily hydrocarbon-contaminated environment (Parks et al. 2017). The abundance of bin 20 was higher in the ferric ion and sulphur-amended samples compared to MA, MCA, MK and MCK. Also, this bacterium had a higher abundance in FA and SA compared to FK and SK. So, bin 20 was positively influenced by the presence of sulphate and ferric ions, which suggested it could utilise them as electron acceptors and/or nutrients. Five other MAGs (bins 01, 04, 09, 12, 21) had elevated abundance in the alkane-amended/biotic control cluster. Bin 01 is a *Thiobacillus* sp., and its closest relative is the *Thiobacillus denitrificans* with 88.72% ANI. This MAG was abundant in the MA, MCA and MK samples, and its incidences were decreased in the FA, SA, FK, SK and MCK samples. Bin 04 was identified as *Methanothrix* sp. with 93.73% ANI to its closest relative *Methanothrix soehngenii*. This archaea might be the main methane producer in the samples, except for MA and MCA where its numbers decreased and bin 11 (*Methanobacterium* sp.) emerged. The abundance of *Methanobacterium* sp. and *Methanothrix* sp. was in an inverse proportionality. Bin 09 is another Actinomycetia bacterium belonging to the UBA2279 family, just as bin 20. Despite the high quality of this MAG (97% completeness and 0.8% contamination), there was no matched relative to it below the family level; therefore, this MAG represents a yet unknown genus. Bin 12 belongs to the genus *Bellilinea* (Chloroflexi) and is related to the *Bellilinea* sp004347695 with 92.51% ANI. This species was found in a 1-methylnaphthalene degrading consortium (Müller et al. 2019). Bin 21 is *G. fermentans* based on the 95.72% ANI. This bin shows a similar abundance pattern to the *Geothrix* in the read based results.

In the aromatics-amended samples, bins 07 and 15 representing the two *Ruminococcus* spp. had high GCPM values compared to their corresponding biotic controls. Bin 20 was also abundant in this cluster of samples, but it had a lower average GCPM compared to the corresponding biotic controls except for MCAR where the abundance equalled to MCK. *F. prausnitzii* (bin 24) also had elevated GCPM values compared to the biotic controls and the alkane-amended samples and showed a similar pattern to the two *Ruminococcus* bins.

There were bins with interesting abundance patterns (Fig. S1). One of them was bin 16 which was only present in the ferric or sulphate ion-amended samples mainly in FAR, SAR, FA, SK and SA. This bin belongs to the Desulfobacterota phylum, and its closest relative is an uncultivated genus CG2-30–66-27 in the MBNT15 family. Bin 14 had an inverse pattern to this, and it was the most abundant in samples MA, MCA, MAR and MCAR. This bin belongs to the SC72 family of Dojkabacteria.

### Functional metagenomics of the hydrocarbon-degrading microbial communities

Eighteen glycyl radical enzymes were found in 11 MAGs. These enzymes may catalyse the activation of hydrocarbon molecules by fumarate addition. Fumarate addition is a frequent way to initiate anaerobic biodegradation of hydrocarbons, and it is carried out by pyruvate-formate lyase (Pfl)-like glycyl radical enzymes (Laczi et al. 2020). Three types of such enzymes have been described so far. Benzyl-succinate synthase (BssA), alkyl-succinate synthase (AssA) and naphthyl-2-methyl succinate synthase (NmsA) catalyse the activation of alkanes, mono- and polyaromatic compounds respectively (Achong et al. 2001; Selesi et al. 2010; Zedelius et al. 2011). The *Cellulomonas* sp. (bin 05) harboured three glycyl radical enzymes belonging to the Pfl-like superfamily. The other *Cellulomonas* MAG (bin 08) harboured two. The two *Ruminococcus* MAGs as well as the *Faecalibacterium prausnitzii* contained one Pfl-like enzyme. Interestingly, bin 23 was only abundant in MA samples and harboured two Pfl-like enzymes. Besides bin 5, bin 23 also may contribute to the hydrocarbon biodegradation in MA samples. In the assembly, we found a gene not binned into the MAGs encoding an enzyme which was highly identical (> 60%) to a proteobacterial NmsA.

The central metabolites of aromatics biodegradation are benzoyl-CoA and its derivatives. There are two benzoyl-CoA-reducing pathways known, ATP dependent and ATP independent (Fuchs et al. 2011; Boll et al. 2014). Bin 16 which was abundant in the aromatics-amended FAR and SAR samples contained a complete set of *bcr* genes encoding the class I type benzoyl-CoA reductase (BCR). This four-subunit enzyme belongs to the BCR/HAD family and is responsible for the ATP-dependent reduction of benzoyl-CoA. We found a similar enzyme set encoded in the two *Ruminococcus* MAGs (bins 07 and 15). In this case, a large enzyme, a fusion of two YjiL ATPase domains and a DUF2229 family domain belonging to the Hgd-D superfamily, were present. Next to this large enzyme coding gene, another Hgd-D-like protein was encoded. The BcrCBAD radical enzyme complex in *T. aromatica* consists of two ATP-binding subunits belonging to the YjiL ATPase family and two Hgd-D-like reductase subunits (Boll et al. 2014; Buckel et al. 2014). This suggests that the large enzyme of the *Ruminococcus* MAGs is an acyl-CoA reductase participating in aromatics biodegradation. The BCR/HAD acyl-CoA reductase family have numerous members with yet unknown functions (Buckel et al. 2014); therefore, the exact function of the ruminococcal acyl-CoA reductase should be investigated. The *Faecalibacterium* MAG also encoded a similar enzyme consisting of one ATP-binding and one CoA-ester binding domain.

Class II type BCRs are responsible for the ATP-independent reduction of benzoyl-CoA. Two MAGs belonging to the UBA1429 order of the Chloroflexota (bins 19 and 22) harboured the full set of *bam*BCDEFGHI-like genes in two gene clusters. Other Chloroflexota MAGs lacked genes, e.g. in bin 13 *bam*D, while in bin 03, *bam*C was missing. Also, in bin 3, the *bam*B-like genes were not clustered with other *bam-*like genes, and in general, *bam*-like genes were scattered in the genome.

## Discussion

In this study, we have investigated the hydrocarbon biodegradation coupled with methanogenesis in consortia derived from chronically contaminated soil.

Methane is a potent energy carrier with a calorific value of approximately 55.5 MJ/kg (Lee et al. 2017). Synthrophic microbes often engage in a mutualist relationship with methanogenic archaea channelling the products of their metabolism into methanogenic pathways (Morris et al. 2013). This phenomenon can be exploited not only in biogas plants but also during the remediation of contaminated sites that lack access to oxygen. Thus, a part of the energy can be recovered from the contaminants. Therefore, we monitored the evolution of methane in our samples on a weekly basis. Except for the abiotic control and the BTEX-containing samples, methane production was observed in all our samples. BTEX compounds in higher than 0.1% (w/v) concentration inhibit the methanogenesis (Siddique et al. 2007) which can explain the lack of methane in the aromatic mixture amended samples. A subset of samples were amended with sulphate or ferric ions to boost microbial activity. We applied them in sufficiently low concentrations to avoid disrupting methanogenesis. The presence of either ferric ions or sulphate increased the methane evolution rate. Methanogenesis and dissimilatory iron reduction are two competing pathways; the latter is more favourable thermodynamically. However, the presence of ferric chloride in low concentrations (less than 200 mg/L) may increase the efficiency of methanogenesis (Siegert et al. 2011; Zhan et al. 2021) instead of decreasing it. In the present experiment, the boosting effect of sulphate and ferric ions on methanogenesis is indirect and may derive from facilitating the metabolism of syntrophic partners. Furthermore, the sulphate reduction may co-exist with methanogenesis, and a low sulphate concentration can increase methane yield (Siegert et al. 2011; Sela-Adler et al. 2017). Carbon dioxide can be utilised in the hydrogenotrophic pathway of methanogenic archaea to yield methane. Certain hydrocarbon degraders like *Smithella* sp. or *Desulfotomaculum* sp. can produce hydrogen during hydrocarbon biodegradation (Abbasian et al. 2015). This hydrogen is used to reduce carbon dioxide to methane. Since *Smithella* sp. was the most abundant in the original soil samples (see the microbial composition and Fig. 4B), we added carbon dioxide to a subset of samples to induce the hydrogenotrophic pathway. However, the methane yield decreased in these samples. High carbon dioxide concentrations may inhibit methanogenesis, but our samples did not contain the high amounts reported previously (Hansson and Molin 1981). Carbon dioxide also can change the preferred methanogenic pathways towards hydrogenotrophy (Mayumi et al. 2013). The GC method for methane measurement separates hydrogen from methane and other gas components. Hence, the presence of hydrogen can be detected in the samples while measuring methane. During the 8 weeks of incubation, hydrogen was not detected in the headspace of the samples which may be explained by the disappearance of *Smithella* sp. The lack of hydrogen impedes the hydrogenotrophic pathway towards which the methanogenesis is driven by the carbon dioxide. Therefore, the methane yield in the carbon dioxide-amended samples decreased.

It is worth mentioning that despite the dynamically increasing methane concentrations in the headspace, the final methane amount remains low, peaking at 37.7 µmol in FK samples at the 8th week of incubation. Relative to 1 g of original soil, this represents approximately 7.5 J of energy. This limits the amount of recoverable energy via methanogenesis-coupled hydrocarbon biodegradation. Therefore, the produced methane can only serve as a cost-reducing agent for bioremediation and is unlikely to generate profit. Collecting the methane is also a challenging and an important task, given methane’s status as a potent greenhouse gas. There are technologies for landfill gas collection that can be adapted; however, their feasibility highly depends on the amounts of methane generated. Some critical parameters like the radius of influence need to be considered while designing such systems (Parameswaran and Sivakumar Babu 2022) as well as the mathematical models for gas generation and migration (Oonk 2012; Majdinasab et al. 2017; Zeng 2020; Huang et al. 2022). The increase in carbon dioxide concentration indicates the metabolic activity of the microbes. In the abiotic controls, there was only a slight increase in CO_2_ concentrations at the beginning without any further CO_2_ evolution. We assume the initial CO_2_ derives from the soil itself. In the aromatics-amended samples, there was a significantly higher CO_2_ production compared to the abiotic controls. However, this increase was only initial and stopped after the first week of incubation. This suggests that the surplus of carbon dioxide is derived from the metabolic activity of the microbial community. Nevertheless, this is just a faint activity compared to the other biotic samples. This indicates that the microbial activity was severely impaired in the presence of toxic compounds, or the aromatic metabolism was stuck at a certain point. The substrates were not converted to CO_2_, or the conversion slowed down somewhere along the pathway.

The biodegradation rate of the aliphatics was the largest in CO_2_-amended samples. A recent study found similar results with resins and asphaltenes when crude oil was degraded by *Klebsiella michiganensis* (Zhang et al. 2023). Carboxylases can be a key factor in the increased biodegradation of alkanes catalysing the conversion of aliphatic compounds into fatty acids (So et al. 2003; Erb 2011; Abbasian et al. 2015). However, some evidence shows this carboxylation is not direct but preceded by a hydroxylation reaction (Callaghan 2013). In the lack of sufficient carbon dioxide, carboxylation processes slow down or stop completely. Hence, the biodegradation rate decreases. In addition, sulphate and ferric ion-amended samples did not fall behind the CO_2_-amended ones, and they are a good alternative for enhancing both methanogenesis and alkane biodegradation via inducing the hydrocarbonoclastic syntrophic bacteria. In addition, the biodegradation of aromatics is also increased by sulphate and ferric ions.

The most abundant genus in the aromatics-amended samples was the *Ruminococcus*. The hydrocarbon-degrading ability of ruminococci was not known so far, although they have been found several times as a dominant genus in hydrocarbon-contaminated samples. Astuti and coworkers reported the emergence of the genus *Ruminococcus* in an oil reservoir after the injection of nutrients, but their prevalence was linked to molasses degradation (Astuti et al. 2022). *Ruminococcus* was the most abundant genus of biofilters used in bioreactors for oil sands process water treatment (Arslan and Gamal El-Din 2021). Another study by Tang and colleagues showed an unidentified Ruminococcaceae bacterium with elevated abundance during PAH biodegradation when the digesters were replenished with straw (Tang et al. 2019). In this case, the strengthening of ruminococci was explained by their cellulolytic activity. In addition, they tolerated the presence of PAHs in the sludge, which is in concordance with our results. Furthermore, the increase in the abundance of the genus *Ruminococcus* along with other acetogenic bacteria was observed in the presence of phenanthrene in the waste-activated sludge (Luo et al. 2016). This suggests that ruminococci not only tolerate PAHs, but the presence of PAHs also positively affects them. However, it remains unclear whether they can utilise aromatics. We have reconstructed two ruminococcal genomes belonging to *R. bicirculans* and *R. bromii*. The presence of glycyl radical enzyme and benzoyl-CoA reductase-like genes in these MAGs further supports their involvement in aromatics biodegradation. Although, it should be mentioned that primary and secondary structure similarities are not sufficient to precisely predict the function of glycyl radical enzymes. This family encompasses a wide variety of enzymes with different activities (ribonucleotide reductases, decarboxylases, formate lyases, x-succinate synthases, and so on) with relatively high similarity to each other (Backman et al. 2017). Therefore, the role of ruminococci in aromatics biodegradation should be further investigated.

The dominance of the genera *Bacteroides* and *Faecalibacterium* in aromatics-amended samples is in concordance with the study of Liu and colleagues in which they observed the *Bacteroides* and *Faecalibacterium* genera becoming dominant in compost treated with 5-ppm benzo[a]pyrene (Liu et al. 2019). The involvement of *Faecalibacterium* in aromatics biodegradation is further supported by the reconstructed MAG of *F. prausnitzii*. Similar glycidyl radical enzyme and benzoyl-CoA coding reductase genes are harboured in it like in the case of the two *Ruminococcus* MAGs.

Another study concluded the involvement of the genus *Bacteroides* in toluene and MTBE removal, although they did not present genomic evidence (Hsia et al. 2021). Unfortunately, we could not recover any MAGs belonging to this genus either. So, the involvement of *Bacteroides* in aromatics biodegradation remains elusive.

Other genera, namely *Clostridium* and *Alistipes*, were also present in aromatics amended samples. *Clostridium* is frequently found as a dominant genus during anaerobic aromatic hydrocarbon degradation (Zhou et al. 2020; Palanisamy et al. 2021; Lv et al. 2022). The genus *Alistipes* was recently shown as a biomarker of hydrocarbon pollution in the intestines of Atlantic cod (Walter et al. 2019), although its role in hydrocarbon biodegradation is unclear. We could not recover any genomes belonging to these genera.

*Cellulomonas* may be involved in alkane biodegradation. *Cellulomonas* was previously reported as a hydrocarbon biodegrader and bioemulsifier producer (Cébron et al. 2022; Chunyan et al. 2023). *Cellulomonas* spp. are facultative anaerobes with the ability to a fermentative lifestyle (Batool et al. 2018) and dissimilatory metal reduction (Sani et al. 2002; Khanal et al. 2021). To our knowledge, anaerobic biodegradation of hydrocarbons by *Cellulomonas* spp. has not been reported yet. In addition, *Cellulomonas* spp. were not just abundant in the aliphatics-amended samples but were also present in the original soil and the enrichment culture as well. Genomic data showed that the two *Cellulomonas* MAGs harbour multiple glycyl radical enzyme genes which can be involved in alkane activation. Therefore, one or more *Cellulomonas* sp. can act as the main hydrocarbon degraders in the aliphatics-amended samples. However, like in the case of *Ruminococcus* and *Faecalibacterium*, their role should be further investigated. We observed the presence of *Actinotalea* in alkane-amended samples. *Actinotalea* has been previously found in various anaerobic hydrocarbon-degrading communities. *Actinotalea* has been suggested to act in the carbon turnover by fermenting carbohydrates and proteins of the dead biomass in the oil-degrading enrichment and providing acetate and carbon dioxide (Li et al. 2019; Semenova et al. 2022). In another study, *Actinotalea* was isolated from underground coal seams and described as a polysaccharide degrader microbe (Vick et al. 2019).

Interestingly, another Actinomicota genus *Streptomyces* was found in the alkane-amended samples and their corresponding biotic controls. *Streptomyces* is a member of normal soil flora capable of hydrocarbon biodegradation, but it utilises the aerobic pathway (Baoune et al. 2019). Some *Streptomyces* spp. are also able to grow under anaerobic conditions (Sangeetha et al. 2022); however, to date, there is no evidence of their ability to utilise hydrocarbons in the absence of oxygen. In contrast, the members of the genus *Microbacterium* can utilise hydrocarbons, mainly PAHs and other aromatics, under anaerobic conditions (Qin et al. 2017; Dhar et al. 2020; Wartell et al. 2021). However, no MAGs were reconstructed from our samples belonging to these genera. In addition, we have observed the elevated abundance of another Actinobacteriota MAG in the presence of alkanes. This indicated the involvement of this bacterium in hydrocarbon biodegradation either as a primary degrader or a syntrophic partner. Although, we could not find in its genome any direct evidence of its involvement in hydrocarbon biodegradation.

The genus *Geothrix* is present in the alkane-amended samples and the biotic controls. We also reconstructed a related MAG and identified as *G. fermentans*. This species was originally isolated from a hydrocarbon-contaminated aquifer (Coates et al. 1999), and their involvement in n-alkane biodegradation was proposed (Rizoulis et al. 2014). However, our genomic data could not support its involvement in anaerobic hydrocarbon biodegradation. On the other hand, *G. fermentans* is an electroactive bacterium which can facilitate interspecies electron transfer and therefor the metabolic activities of other microbes (Aulenta et al. 2020; Conners et al. 2022).

Many Chloroflexota MAGs have been recovered showing different patterns of abundance among the samples. Members of Chloroflexi were proposed to be scavengers of necromass in hydrocarbon-degrading microbiomes (Tan et al. 2015). A recent study showed metatranscriptomic evidence of *Bellilinea* sp. utilising dead biomass in hydrocarbon-degrading anaerobic cultures (Liu et al. 2022). The recovered MAGs showed evidence of the capability of Chloroflexota to degrade benzoyl-CoA which is a central metabolite of aromatics biodegradation. Based on our results, bacteria belonging to the phylum Chloroflexota utilise the ATP-independent benzoyl-CoA reduction pathway. Therefore, they can be involved in aromatics biodegradation.

We also found some MAGs with interesting abundance patterns. One of them belongs to the MBNT15 bacterial group. The members of MBNT15 were first observed in a boreal peatland microbiome. They were capable of dissimilatory iron reduction and were proposed to be scavengers (Begmatov et al. 2022). The other belongs to the Dojkabacteria which was first observed in hydrocarbon and chlorinated solvent-contaminated aquifers by Dojka and colleagues (Dojka et al. 1998). Then, it was named after their discoverer by Wrighton and colleagues (Wrighton et al. 2016). Members of this taxon are capable of CO_2_ fixation via the pentose bisphosphate pathway. This could be an explanation for bin 14 having a high abundance in MCA and MCAR samples, but its numbers were low in MCK. We could observe a similar pattern in MA, MAR and MK. In MAR, we did not detect any CO_2_ evolution during the incubation period, and MK had similar CO_2_ levels as MA. This suggested that CO_2_ fixation was not the main route for this bacterium in our samples, but it directly or indirectly participated in hydrocarbon biodegradation for its high abundance is linked to the additional carbon source amended samples.

Methanogenic archaea are important in syntrophic relationships channelling the electrons derived from the metabolic pathways of syntrophic partners towards methane generation (Leahy et al. 2022). Thus, they are the key actors in methanogenesis-coupled hydrocarbon biodegradation. In the majority of our samples, *Methanothrix* sp. is the most abundant. The bulk of the members of this genus are strictly acetoclastic (Khanh Nguyen et al. 2021). In addition, Holmes and colleagues showed metatranscriptomic evidence that *Methanothrix* sp. can accept electrons via the conductive pili of *Geobacter* (Holmes et al. 2017; Zhou et al. 2023). *Methanobacterium* sp. is another methanogenic archaea found in our samples. The members of this genus utilise the hydrogenotrophic pathway to produce methane (Khanh Nguyen et al. 2021). In contrast to *Methanothrix*, *Methanobacterium* has a lower abundance suggesting that *Methanothrix* and the acetotrophic pathway are responsible for the majority of the methane produced. This is also supported by the reduced methane evolution rate in the presence of carbon dioxide. In addition, DIET between *Methanothrix* and the exoelectrogenic hydrocarbon degrader *Geothrix* is also possible.

In conclusion, we have found that the addition of sulphate and ferric ions in low concentrations facilitate both the methanogenesis and the hydrocarbon biodegradation. The energy recovered in the form of methane is relatively low and only suitable for eventually decreasing rehabilitation costs; however, the contaminated site must be thoroughly investigated, and the gas collection system must be carefully designed prior to bioremediation. Whole metagenomic analysis pinpointed some new candidates for hydrocarbon biodegradation (*Cellulomonas*, *Ruminococcus*, *Faecalibacterium*), while the members of Chloroflexota can act as secondary degraders. Analysing their recovered genomes, we have identified genes coding for glycyl radical enzymes, which may function as alkyl- or benzylsuccinate synthases, and types I and II benzoyl-CoA reductases; however, their roles must be clarified in future studies.

## Supplementary Information

Below is the link to the electronic supplementary material.

Supplementary file1 (PDF 172 KB)

## Data Availability

Raw sequencing data were uploaded to the European Nucleotide Archive under the project ID PRJEB15034.
