# Supplementary material for: Methanogenesis coupled hydrocarbon biodegradation enhanced by ferric and sulphate ions
Source: Appl Microbiol Biotechnol. 2024 Aug 29;108:449. doi: 10.1007/s00253-024-13278-0 (PMC11362221; doi:10.1007/s00253-024-13278-0)
Supplement: Supplementary file 1 [file 253_2024_13278_MOESM1_ESM.pdf]

Applied Microbiology and Biotechnology

Methanogenesis coupled hydrocarbon biodegradation enchanced by ferric and sulfate ions

Krisztián Laczi[1,2,*], Attila Bodor[1,3], Tamás Kovács[4], Balázs Magyar[5], Katalin Perei[1,#], Gábor Rákhely[1,3,#,†]

[1]Department of Biotechnology, University of Szeged, Szeged, Hungary

[2]Institute of Plant Biology, Biological Research Centre, Hungarian Research Network, Szeged, Hungary

[3]Institute of Biophysics, Biological Research Centre, Hungarian Research Network, Szeged, Hungary

[4]Department of Biotechnology, Nanophagetherapy Center, Enviroinvest Corporation, Pécs, Hungary

[5]Biocentrum Ltd., Gyöngyösoroszi, Hungary

* Correspondence to: 52 Közép fasor, room 148, Szeged, Hungary, e-mail address: laczi.krisztian@bio.u-szeged.hu

[#]These authors contributed equally

†Gábor Rákhely has passed away on 04.11.2023.

Table S1: Energy content of the produced methane				
Sample	Total Energy Content of the Produced Methane		Energy /gramm original soil	
	Energy (J)	Standard deviation	Energy (J)	Standard deviation
MA	21.64472	7.330289	4.809939	1.628953
MCA	5.665683	2.303133	1.259041	0.511807
MK	19.56133	7.737779	4.346963	1.719506
MCK	8.091811	5.234096	1.79818	1.163132
FA	30.40453	6.123205	6.756563	1.360712
FK	33.54919	3.668899	7.455376	0.815311
SA	27.60783	2.940144	6.135074	0.653365
SK	17.99118	4.056786	3.99804	0.901508

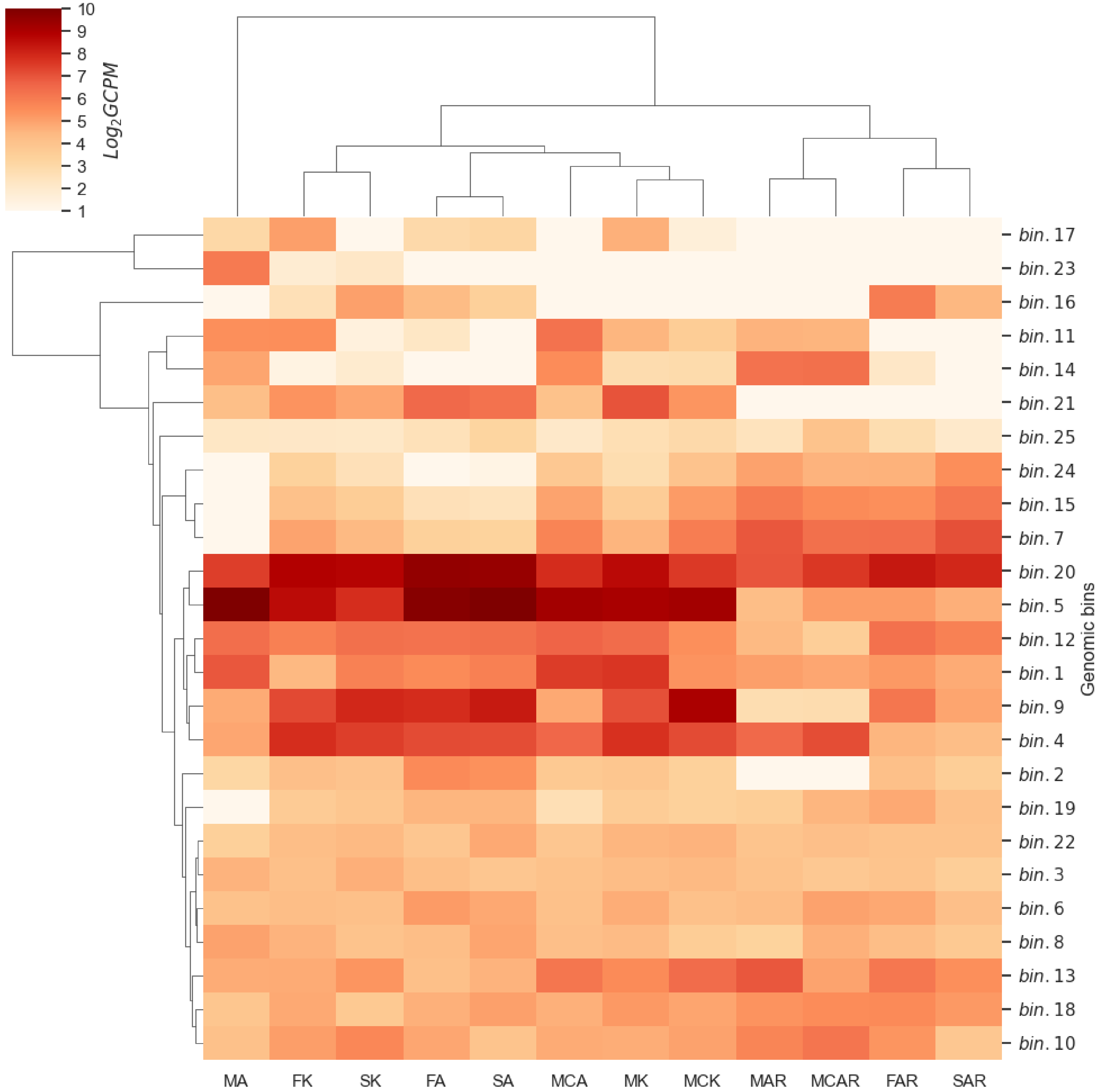

Supplementary Figure 1. Cluster map of the bin abundances across the samples. Bin abundance was calculated with MetaWrap's Quant_bins module. The reads of individual samples were mapped back to the bins. The abundance of the bins described in GCPM was calculated from the length-weighted abundance of the contigs by salmon. The average $\log_2$ GCPM values were visualized with the cluster map module of Seaborn using the Bray-Curtis method.
